# Support Vector Machine Model Predicts Dose for Organs at Risk in High-Dose Rate Brachytherapy of Cervical Cancer

**DOI:** 10.3389/fonc.2021.619384

**Published:** 2021-07-15

**Authors:** Ping Zhou, Xiaojie Li, Hao Zhou, Xiao Fu, Bo Liu, Yu Zhang, Sheng Lin, Haowen Pang

**Affiliations:** ^1^ Department of Radiology, The Affiliated Hospital of Southwest Medical University, Luzhou, China; ^2^ Department of Oncology, The Affiliated Hospital of Southwest Medical University, Luzhou, China; ^3^ Department of Nursing College, Southwest Medical University, Luzhou, China

**Keywords:** brachytherapy, cervical cancer, organs at risk, support vector machine, dose prediction

## Abstract

**Introduction:**

This study aimed to establish a support vector machine (SVM) model to predict the dose for organs at risk (OARs) in intracavitary brachytherapy planning for cervical cancer with tandem and ovoid treatments.

**Methods:**

Fifty patients with loco-regionally advanced cervical cancer treated with 200 CT-based tandem and ovoid brachytherapy plans were included. The brachytherapy plans were randomly divided into the training (N = 160) and verification groups (N = 40). The bladder, rectum, sigmoid colon, and small intestine were divided into sub-OARs. The SVM model was established using MATLAB software based on the sub-OAR volume to predict the bladder, rectum, sigmoid colon, and small intestine ${\text{D}}_{2\text{c}{\text{m}}^{3}}$. Model performance was quantified by mean squared error (MSE) and δ $\left(\delta =\left|{\text{D}}_{2\text{c}{\text{m}}^{3}}/{\text{D}}_{\text{prescription}}\left(\text{actual}\right)-{\text{D}}_{2\text{c}{\text{m}}^{3}}/{\text{D}}_{\text{prescription}}\left(\text{predicted}\right)\right|\right)$. The goodness of fit of the model was quantified by the coefficient of determination (R^2^). The accuracy and validity of the SVM model were verified using the validation group.

**Results:**

The ${\text{D}}_{2\text{c}{\text{m}}^{3}}$ value of the bladder, rectum, sigmoid colon, and small intestine correlated with the volume of the corresponding sub-OARs in the training group. The mean squared error (MSE) in the SVM model training group was <0.05; the R^2^ of each OAR was >0.9. There was no significant difference between the ${\text{D}}_{2\text{c}{\text{m}}^{3}}$ -predicted and actual values in the validation group (all *P* > 0.05): bladder δ = 0.024 ± 0.022, rectum δ = 0.026 ± 0.014, sigmoid colon δ = 0.035 ± 0.023, and small intestine δ = 0.032 ± 0.025.

**Conclusion:**

The SVM model established in this study can effectively predict the ${\text{D}}_{2\text{c}{\text{m}}^{3}}$ for the bladder, rectum, sigmoid colon, and small intestine in cervical cancer brachytherapy.

## Introduction

Cervical cancer is the most common malignancy among women in developing countries (1). Depending on the stage of diagnosis, the treatment strategies for cervical cancer mainly include surgery, along with radiotherapy and chemotherapy (2). For patients with locally advanced cervical cancer, brachytherapy combined with external-beam radiotherapy is the prevalent standard treatment (3). Three-dimensional brachytherapy is widely applied in clinical practice, and computed tomography (CT)- or magnetic resonance imaging (MRI)-based treatment planning systems (TPS) provide accurate tumor and organs at risk (OARs) dose information. However, the experience of brachytherapy planners and knowledge of the Radiation Therapy Oncology Group guidelines, as well as clinical expertise and intuition, have a significant effect on the quality of a brachytherapy plan (4). If a planner can predict the OAR dose before designing a brachytherapy plan, the quality of the brachytherapy plan can be controlled, and the interfering factors can be minimized. Previous reports on cervical cancer brachytherapy have described the effects of the volume of the OARs on the dose to the bladder, rectum, sigmoid colon, and small intestine (5). Although there is a correlation between the dose to the OARs and their volumes, information to predict the dose to the OARs is limited. In recent years, methods for predicting the dose to the OARs have been widely introduced in external irradiation intensity-modulated radiotherapy (6–10). These approaches typically use libraries of existing patient plans to create models that predict the extent of OAR sparing that can be achieved in a new patient based on, for example, the planning target volume (PTV)-OAR distance and overlap (11). In this study, we examined factors relevant for the dose to the bladder, rectum, sigmoid colon, and small intestine in cervical cancer brachytherapy based on the Fletcher applicator. The bladder, rectum, sigmoid colon, and small intestine were divided into sub-OARs. We analyzed the correlation between the sub-organ volume and ${\text{D}}_{2\text{c}{\text{m}}^{3}}$ of each OAR, and the SVM prediction model based on the correlation was established to predict the dose of each OAR before brachytherapy; the model can be used as an evaluation standard for brachytherapy plans to minimize the effects of confounding factors on the quality of the plans. To our knowledge, this study is the first to apply the SVM model to OAR dosimetric prediction based only on the contours of the organs and targets. This approach has been granted a Chinese invention patent (patent no.: 201610529290.8).

## Materials and Methods

### Patients

We retrospectively selected 50 patients with loco-regionally advanced cervical cancer treated with 200 CT-based tandem and ovoid brachytherapy plans between 2016 and 2018 in the Affiliated Hospital of Southwest Medical University. The patients treated with brachytherapy were randomly divided into the training (N = 160) and verification groups (N = 40). The cervical cancer stages ranged from ІІB to IVA, according to the International Federation of Gynecology and Obstetrics system.

### Targets and Delineation of the OARs

The high-risk clinical target volume (HR-CTV) contours were generated for each treatment based on the Gynaecological European Society for Radiotherapy and Oncology Working Group I (Gyn GEC-ESTRO WG I) recommendations (12). The HR-CTV covered the entire cervix and macroscopic extent of the disease, based on clinical examinations and as depicted in CT images. The OARs included the bladder, rectum, sigmoid colon, and small intestine. The same radiation oncologist performed the target and delineation of the OARs.

### Prescription Dose and Limiting Requirements for the OARs

After receiving 45 Gy intensity-modulated radiation therapy (IMRT), the per fraction prescription dose (D_prescription_) for the HR-CTV was defined as 7 Gy with a total of four fractions for brachytherapy. A prescription dose delivered to 90% of the HR-CTV was considered. Combined with the IMRT dose, the total EQD2 (equivalent dose in 2 Gy, α/β = 10) for HR-CTV and IR-CTV was 85 and 60 Gy, respectively. We applied dose constraints for the OARs according to the following principles: combined IMRT dose, ${\text{D}}_{2\text{c}{\text{m}}^{3}}$ of EQD2 of ≤90 Gy (α/β = 3) for bladder, ≤75 Gy (α/β = 3) for rectum, ≤75 Gy (α/β = 3) for sigmoid colon, and ≤75 Gy (α/β = 3) for small intestine. These dose constraints were primarily based on the Gynaecological European Society for Radiotherapy and Oncology Working Group II (Gyn GEC-ESTRO WG II) recommendations (13). The ^192^Ir-source was delivered using the Fletcher applicator. To avoid bladder and rectum volume variations, the bladder of all patients was emptied and subsequently filled with 50 ml of saline solution; they accepted an enema to empty the rectum before brachytherapy.

### Brachytherapy Plans

The Oncentra 4.3 treatment planning system (Elekta Brachytherapy, Veenendaal, the Netherlands) was used for the brachytherapy plans. All brachytherapy plans in this study were developed using a manual and/or graphical optimization approach to repeatedly optimize the plan and thus ensure that the dose administered to 90% of the HR-CTV reached the prescribed dose (D_prescription_), whereas the dose to the OARs was lower. For the optimization of the single brachytherapy plan, the prescription dose (7 and 4.2 Gy) was administered to 90% of HR-CTV and IR-CTV; ${\text{D}}_{2\text{c}{\text{m}}^{3}}$ of the bladder < 5.2 Gy, ${\text{D}}_{2\text{c}{\text{m}}^{3}}$ of the rectum, sigmoid colon, and small intestine < 4.7 Gy.

### Deriving Sub-OARs From the OARs

**Figure 1:**
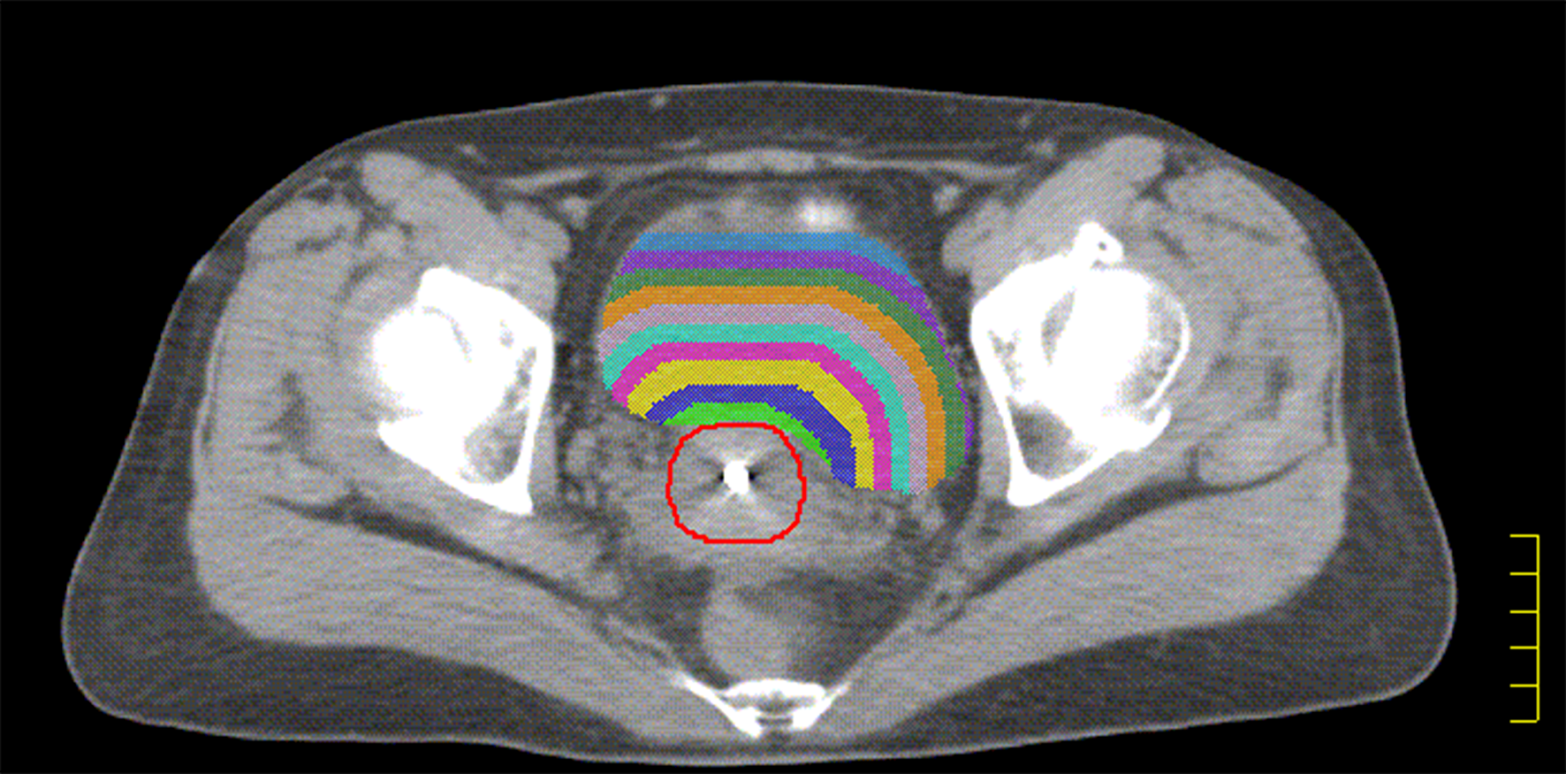
The red line indicated HR-CTV, The green shadow indicated the intersection of ring_1_ and bladder, the blue shadow indicated the intersection of ring_2_ and bladder. The yellow shadow indicated the intersection of ring_3_ and bladder. The purple shadow indicated the intersection of ring_4_ and bladder. The sky blue shadow indicated the intersection of ring_5_ and bladder. The lavender shadow indicated the intersection of ring_6_ and bladder. The orange shadow indicated the intersection of ring_7_ and bladder. The forest shadow indicated the intersection of ring_8_ and bladder. The slate blue shadow indicated the intersection of ring_9_ and bladder.

The HR-CTV was externally expanded to a plurality of rings (ring1–ringn) with a width of 0.5 cm using the Oncentra 4.3 treatment planning system. Ring1–ringn and different OAR intersection regions (ring1–ringn∩OAR) were used as independent sub-OARs, with ring1∩OAR defined as the sub-OAR1, and so on; ringn∩OAR was defined as sub-OARn. The total sub-OARs are controlled within 10 and the statistics of the volume of each sub-OARs. The intersecting regions for ring1–ring9 and the bladder in patient 15 are shown in **Figure 1**.

### SVM Model Development

In machine learning, support vector machine (SVM) are supervised learning models with the associated learning algorithms used to analyze data for classification and regression analysis. In our study, we applied a radial basis function kernel for binary classification. We used MATLAB (R2017a, MathWorks, Inc., Natick, MA, USA) software to read, prepare, process, and output the predicted value. The SVM models were trained, validated, and tested for prediction accuracy using a self-written algorithm in MATLAB. A common radial basis function kernel was used:

$$K(x_{i},x_{j})=e^{{\left(-\gamma \left||x_{i}-x_{j}|\right|\right)}^{2}}$$

where x_i_ and x_j_ are two data points, and γ is the shape parameter that represents the equivalent to the standard deviation in Gaussian distribution. To deal with the problem of regularization for noisy data, a user-specified cost parameter C is introduced, which acts to soften the margin. The cost parameter C controls the trade-off between allowing transgression of data points across the margin edges toward the other class and a more complex boundary, which might lead to overfitting. The evaluation and choice of C and γ were conducted using a grid search. The optimal parameters were estimated using the training and validation sets. We analyze the correlation between the sub-organ volume and ${\text{D}}_{2\text{c}{\text{m}}^{3}}$ of each OAR and establish the SVM prediction model based on the correlation. The volumes of the sub-OARs were used as the independent variable in the SVM model, and the ${\text{D}}_{2\text{c}{\text{m}}^{3}}/{\text{D}}_{\text{prescription}}$ ratios were used as the dependent variable.

For the verification group, the performance of the SVM model was investigated to predict ${\text{D}}_{2\text{c}{\text{m}}^{3}}/{\text{D}}_{\text{prescription}}$ per fraction in the bladder, rectum, sigmoid, and small intestine using the volumes of the corresponding sub-OARs. The volumes of the sub-OARs were used as the input values for the SVM model, and the ${\text{D}}_{2\text{c}{\text{m}}^{3}}/{\text{D}}_{\text{prescription}}$ ratios were used as the output values. The performance of the model can be characterized by mean squared error (MSE) and δ $\left(\delta =\left|{\text{D}}_{2\text{c}{\text{m}}^{3}}/{\text{D}}_{\text{prescription}}\text{(actual)}-{\text{D}}_{2\text{c}{\text{m}}^{3}}/{\text{D}}_{\text{prescription}}\left(\text{predicted}\right)\right|\right)$. The goodness of fit of the model was quantified by the coefficient of determination (R^2^ = 1 − the ratio of the sum of squares regressed to the total sum of squares). R^2^ indicates the proportionate amount of variation in the response variable explained by the independent variables in the model. They measure the fitting performance of a model from different perspectives. The closer the δ is to 0, the closer the actual and prescription values are to each other. Furthermore, the closer the R^2^ is to 1, the higher the fitting degree.

### Statistical Analysis

Significant differences were determined using a two-sided paired *t*-test with SPSS 19.0 software (SPSS, Inc., Chicago, IL, USA). Correlations were tested by performing the Pearson correlation coefficient analysis. P <0.05 indicates that there is a correlation between the two variables, and P <0.01 indicates that there is a significant correlation between the two variables.

## Results

The volume of each sub-OAR (V_sub-OAR_) was correlated with the ${\text{D}}_{2\text{c}{\text{m}}^{3}}/{\text{D}}_{\text{prescription}}$ of the respective OAR. The volume of the HR-CTV (V_HR-CTV_) was correlated with the ${\text{D}}_{2\text{c}{\text{m}}^{3}}/{\text{D}}_{\text{prescription}}$ of the bladder, rectum, and sigmoid colon (all correlations, *P* < 0.05). The volume of the bladder (V_bladder_) and the ${\text{D}}_{2\text{c}{\text{m}}^{3}}/{\text{D}}_{\text{prescription}}$ of the small intestine were correlated. The correlation coefficient (*r*, a statistical index used to describe the degree of linear correlation between two variables), and *P* values are shown in **Tables 1**–**4**. Therefore, these data can be used to predict the ${\text{D}}_{2\text{c}{\text{m}}^{3}}/{\text{D}}_{\text{prescription}}$ of each OAR using the SVM model. The MSE and the R^2^ of each OAR in the SVM model prediction group are shown in **Table 5**.

**Table 1:** The correlation coefficient (r) and *P* value for bladder.

Relevant factors r, *P*	VHR-CTV	Vsub-bladder 1	Vsub-bladder 2	Vsub-bladder 3	Vsub-bladder 4	Vsub-bladder 5
$\text{r}({\text{D}}_{2\text{c}{\text{m}}^{3}}/{\text{D}}_{\text{prescription}})$	0.45**	0.58**	0.49**	0.45**	0.41**	0.37**
$P({\text{D}}_{2\text{c}{\text{m}}^{3}}/{\text{D}}_{\text{prescription}})$	0.001	<0.001	<0.001	0.001	0.003	0.008

**When the confidence (double test) is less than 0.01, the correlation is significant.

Vsub-bladder, the volume of the sub-bladder.

**Table 2:** The correlation coefficient (r) and *P* value for rectum.

Relevant factors r, *P*	VHR-CTV	Vsub-rectum 1	Vsub-rectum 2	Vsub-rectum 3	Vsub-rectum 4	Vsub-rectum 5
$\text{r}({\text{D}}_{2\text{c}{\text{m}}^{3}}/{\text{D}}_{\text{prescription}})$	0.40**	0.59**	0.37**	0.36**	-0.29*	-0.28*
$P({\text{D}}_{2\text{c}{\text{m}}^{3}}/{\text{D}}_{\text{prescription}})$	0.005	<0.001	0.009	0.009	0.028	0.033

**When the confidence (double test) is less than 0.01, the correlation is significant, *when the confidence (double test) is less than 0.05, the correlation is significant, the negative indicates that there is a negative correlation between the Vsub- rectum and ${\text{D}}_{2\text{c}{\text{m}}^{3}}/{\text{D}}_{\text{prescription}}$ of rectum.

Vsub-rectum, the volume of the sub-rectum.

**Table 3:** The correlation coefficient (r) and *P* value for sigmoid colon.

Relevant factors r, *P*	VHR-CTV	Vsub-sigmoid colon 1	Vsub-sigmoid colon 2	Vsub-sigmoid colon 3	Vsub-sigmoid colon 4	Vsub-sigmoid colon 5
$\text{r}({\text{D}}_{2\text{c}{\text{m}}^{3}}/{\text{D}}_{\text{prescription}})$	0.36**	0.85**	0.90**	0.85**	0.54**	0.57**
$P({\text{D}}_{2\text{c}{\text{m}}^{3}}/{\text{D}}_{\text{prescription}})$	0.010	<0.001	<0.001	<0.001	<0.001	<0.001

**When the confidence (double test) is less than 0.01, the correlation is significant.

Vsub-sigmoid colon, the volume of the sub-sigmoid colon.

**Table 4:** The correlation coefficient (r) and *P* value for small intestine.

Relevant factors r, *P*	VBladder	Vsub-small intestine 1	Vsub-small intestine 2	Vsub-small intestine 3	Vsub-small intestine 4	Vsub-small intestine 5
$\text{r}({\text{D}}_{2\text{c}{\text{m}}^{3}}/{\text{D}}_{\text{prescription}})$	0.75**	0.89**	0.89**	0.87**	0.84**	0.83**
$P({\text{D}}_{2\text{c}{\text{m}}^{3}}/{\text{D}}_{\text{prescription}})$	<0.001	<0.001	<0.001	<0.001	<0.001	<0.001

**When the confidence (double test) is less than 0.01, the correlation is significant.

Vsub-small intestine, the volume of the sub-small intestine.

**Table 5:** The MSE and the r-squared of each OAR for the SVM prediction model group.

	Bladder	Rectum	Sigmoid colon	Small intestine
MSE	0.00270	0.00024	0.00104	0.00102
R^2^	0.938	0.991	0.957	0.964

MSE, mean squared error; SVM, support vector machine; R^2^, the coefficient of determination.

**Figure 2:**
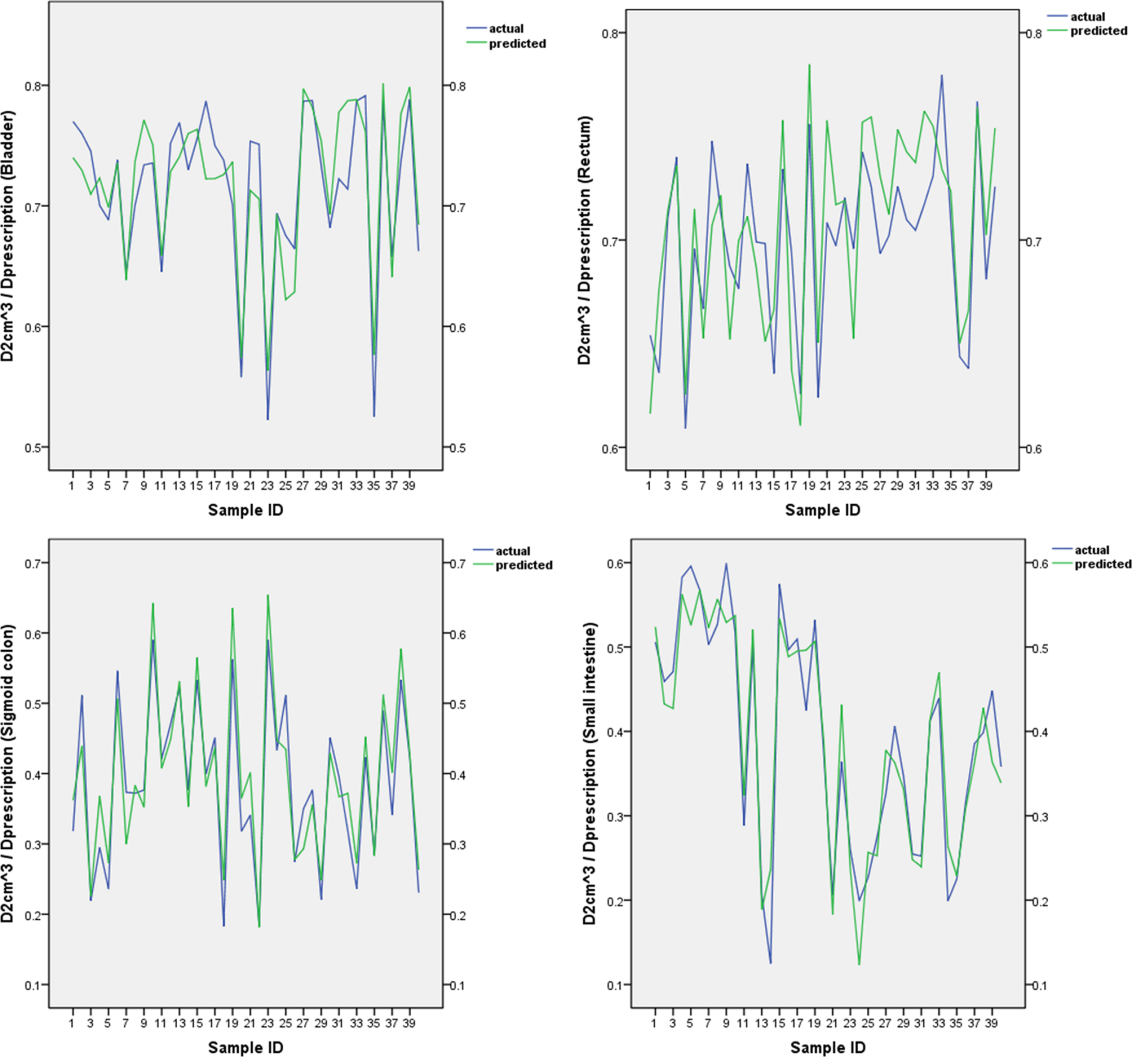
The predicted and actual ${\text{D}}_{2\text{c}{\text{m}}^{3}}/{\text{D}}_{\text{prescription}}$ values for the bladder, rectum, sigmoid colon, and small intestine.

The predicted and actual ${\text{D}}_{2\text{c}{\text{m}}^{3}}/{\text{D}}_{\text{prescription}}$ values for the bladder, rectum, sigmoid colon, and small intestine in the validation group are shown in **Figure 2**. There was no statistically significant difference between the predicted and actual ${\text{D}}_{2\text{c}{\text{m}}^{3}}/{\text{D}}_{\text{prescription}}$ values for the bladder (*P =* 0.68), rectum (*P =* 0.16), sigmoid colon (*P =* 0.14), and small intestine (*P =* 0.77) in the validation group. The δ value for the bladdera of the verification group was 0.024 ± 0.022, the corresponding rectum δ value was 0.026 ± 0.014, the sigmoid colon δ value was 0.035 ± 0.023, and the small intestine δ value was 0.032 ± 0.025.

## Discussion

The quality control of radiotherapy plan has always been a research hotspot in the field of radiotherapy (14–17). The most critical aspect is the prediction of the dose to the OAR before designing the radiotherapy plan. It has been reported that the OAR dose in the brachytherapy plan could be predicted by the overlapping volume of the OAR with the targeted area and knowledge-based tool (18, 19). Ours is a relatively simple mathematical model that uses prescription dose and V_sub-OAR_ to predict the bladder, rectum, and sigmoid ${\text{D}}_{2\text{c}{\text{m}}^{3}}$ for brachytherapy; this does not require buying new modules of TPS or extracting the distance of each sampling point of the OAR with the dose. We also divided the OARs into multiple sub-OARs to predict the OAR dose in the external IMRT plan (20, 21). In contrast to previous studies, the focus of our study is to determine the correlation between the V_sub-OAR_ and ${\text{D}}_{2\text{c}{\text{m}}^{3}}/{\text{D}}_{\text{prescription}}$ of each OAR in brachytherapy, therefore, this method has been granted the Chinese invention patent. Owing to this correlation, we could fit the data of the training group using the SVM model approach. To rule out the effects of different prescription doses on the ${\text{D}}_{2\text{c}{\text{m}}^{3}}$ of each OAR, we divided each ${\text{D}}_{2\text{c}{\text{m}}^{3}}$ by 90% of the HR-CTV that reached the dose (D_prescription_).

As shown in **Figure 2**, our SVM estimation system predicted that the ${\text{D}}_{2\text{c}{\text{m}}^{3}}/{\text{D}}_{\text{prescription}}$ of the OARs is very close to the actual value. There was no significant difference between the predicted and actual ${\text{D}}_{2\text{c}{\text{m}}^{3}}/{\text{D}}_{\text{prescription}}$ values for each OAR. The δ values of the bladder, rectum, sigmoid colon, and small intestine were 0.024 ± 0.022, 0.026 ± 0.014, 0.035 ± 0.023, and 0.032 ± 0.025, respectively. The abovementioned statistics, MSE, and R^2^ of the SVM prediction model indicated that the prediction model was reliable. We used a relatively simple mathematical model, which does not require the acquisition of new modules of TPS software. The process model for the acquisition of the sub-OAR can be edited into scripts to improve efficiency and effectiveness.

Our model can be used as a component of a quality assurance tool to detect suboptimal treatment plans in OAR sparing. A properly trained model will provide an estimate of the OAR doses required for appropriate planning and will detect outlines that require further review. Specifically, considering δ, a δ value closer to 0 indicated a closer relationship between the planned and predicted values of ${\text{D}}_{2\text{c}{\text{m}}^{3}}/{\text{D}}_{\text{prescription}}$. A standard δ threshold can be set for the ${\text{D}}_{2\text{c}{\text{m}}^{3}}$ of each OAR, and the value above the threshold should be further optimized or the position of the applicator should be re-adjusted, until a satisfactory δ value is obtained. Predictions using the SVM model can be conducted for the quality control of the brachytherapy plan and for minimizing the effect of subjective factors (22).

Our study has some limitations. It was restricted to a single institution and considered only standard tandem and ovoid cases. Further research is needed comprising multiple centers and more cervical cancer brachytherapy plan data sets for analysis. If the data set is large enough, a neural network model can be developed, which will generate predictions with higher accuracy of the OAR dose for cervical cancer brachytherapy plans. The SVM models discussed herein may be applied beyond gynecologic brachytherapy. The application of our models to prostate brachytherapy as well can be considered after validation.

## Conclusion

The SVM model can be applied to not only predict the dose to the OARs for the high-dose rate brachytherapy of cervical cancer but also develop quality assurance tools for designing brachytherapy plans.

## Data Availability Statement

The raw data supporting the conclusions of this article will be made available by the authors, without undue reservation.

## Ethics Statement

The studies involving human participants were reviewed and approved by The Affiliated Hospital of Southwest Medical University Ethics Committee. The patients/participants provided their written informed consent to participate in this study.

## Author Contributions

Guarantors of integrity of entire study, HP. Study concepts/study design or data acquisition or data analysis/interpretation, all authors. Manuscript drafting or manuscript revision for important intellectual content, all authors. Resolution of any questions related to the work, all authors. Literature research, PZ, SL, and HP. Statistical analysis, PZ, XL, and HP. Manuscript editing, PZ, SL, and HP. All authors contributed to the article and approved the submitted version.

## Conflict of Interest

The authors declare that the research was conducted in the absence of any commercial or financial relationships that could be construed as a potential conflict of interest.
